# A latent profile analysis of self-management behavior among patients after metabolic bariatric surgery

**DOI:** 10.3389/frhs.2026.1774099

**Published:** 2026-03-13

**Authors:** Wenbin He, Ying Zhang, Li Du, Jian Yang, Ying Wang, Bilong Feng

**Affiliations:** 1 Department of Colorectal and Anal Surgery, Zhongnan Hospital of Wuhan University, Wuhan, China; 2 Health Science Center, Yangtze University, Jingzhou, China; 3 Department of Nursing, Zhongnan Hospital of Wuhan University, Wuhan, China; 4 Hepatobiliary & Pancreatic Surgery, Zhongnan Hospital of Wuhan University, Wuhan, China; 5 Thoracic Surgery, Zhongnan Hospital of Wuhan University, Wuhan, China; 6 Department of Infection Prevention and Control Management, Zhongnan Hospital of Wuhan University, Wuhan, China; 7 Hubei Engineering Center for Infectious Disease Prevention, Control and Treatment, Wuhan, China

**Keywords:** latent profile analysis, metabolic bariatric surgery, obesity, self-efficacy, self-management

## Abstract

**Introduction:**

Obesity has emerged as a global public health epidemic with far-reaching health consequences. While metabolic bariatric surgery (MBS) is an established therapeutic modality for moderate-to-severe obesity and associated metabolic disorders, enabling rapid weight reduction and metabolic improvement, postoperative weight regain remains a critical barrier to sustaining long-term treatment efficacy. Indeed, the durability of surgical outcomes is heavily contingent upon patients’ ability to engage in sustained self-management behaviors.

**Aims:**

To characterize the patterns of self-management behavior among patients after metabolic bariatric surgery using latent profile analysis, and to examine the relationships among these latent profiles.

**Methods:**

A cross-sectional study was carried out at one general hospital. A total of 242 patients after metabolic bariatric surgery completed the socio-demographic questionnaire, Bariatric Surgery Self-Management Questionnaire, General Self-efficacy Scale, and International Physical Activity Questionnaire.

**Results:**

Three latent profiles were identified: high self-management behavior group (*n* = 28, 11.57%), moderate self-management behavior group (*n* = 156, 64.46%) and low self-management behavior group (*n* = 58, 23.97%). The ANOVA and chi-square tests demonstrated significant differences among three groups concerning age and educational level. Multinomial logistic regression analysis indicated that employment condition, smoking and drinking history significantly predicted self-management behavior.

**Conclusions:**

The statistical analysis indicated that the majority of patients fall into the moderate self-management group. Further regression analysis demonstrated significant associations between self-management proficiency and both age and educational level. These findings highlight the need for tailored interventions targeting specific patient profiles.

## Introduction

Obesity has emerged as a pressing global public health challenge, affecting populations across all demographics and throughout the entire life cycle (1). As an independent chronic disease with distinct pathophysiological characteristics, obesity is not only pathologically significant in its own right but also recognized as an independent risk factor for chronic non-communicable diseases that severely threaten human health, including cardiovascular disorders, type 2 diabetes, and certain malignancies (2, 3). In the context of clinical intervention for obesity, the American Society for Metabolic and Bariatric Surgery (ASMBS) and the International Federation for the Surgery of Obesity and Metabolic Disorders (IFSO) have jointly established clinical guidelines specifying that metabolic bariatric surgery may be considered as a treatment option for: (1) obese patients with a body mass index (BMI) ≥ 32.5 kg/m^2^; or (2) those with a BMI ≥30 kg/m^2^ complicated by obesity-related comorbidities such as metabolic syndrome or T2DM, when conventional medical treatments have failed to produce adequate weight loss or metabolic improvement (4). MBS achieves weight regulation through surgical approaches and has been clinically validated to effectively reduce body weight while significantly lowering the risk of obesity-related complications (5). According to the latest statistics from IFSO, over 500,000 MBS procedures are performed worldwide annually (6). In the Chinese population, the clinical application of metabolic bariatric surgery has also shown a rapid growth trend. According to the China obesity and metabolic surgery database, the total number of metabolic bariatric surgeries in China was approximately 37,249 in 2023 (7). Relevant studies have shown that over 60% of postoperative patients experience varying degrees of weight regain within 3 to 10 years, and this weight gain is often accompanied by the recurrence of obesity-related complications (8). A Chinese cohort study identified that 16.6%–46.6% of patients after MBS engaged in “herding” eating behaviors, and among these individuals, 47% experienced weight regain post-surgery (9). Notably, the long-term maintenance of optimal weight loss outcomes post-surgery heavily relies on sustained improvements in dietary patterns and exercise habits, the success of which largely depends on individuals’ self-health management capabilities.

Robust self-management capabilities are widely recognized as critical for patients following metabolic bariatric surgery, as they play a pivotal role in mitigating nutrient deficiencies, reducing complication risks, and preventing postoperative weight regain (10, 11). Nevertheless, prior research highlights prevalent challenges in this population, including suboptimal adherence to dietary and exercise regimens, emotional eating patterns, and inadequate trace element intake (12, 13). For example, Welch et al.'s seminal work demonstrated that overall behavioral adherence among patients after MBS was moderate, with dietary practices and physical activity exhibiting the lowest adherence rates (14). Importantly, this study also established that higher adherence levels correlated with more pronounced postoperative weight reduction and improved nutritional status—findings that align with subsequent observations by Marek (15). Complementing these insights, Sobhani demonstrated that self-management capacity exhibited a positive correlation with anthropometric outcomes: patients with high self-management behaviors showed greater improvements in key metrics such as body weight, waist circumference, and BMI (16). These findings underscore the necessity of close monitoring and targeted management of patients’ self-management behaviors to optimize and sustain the long-term efficacy of MBS.

Scholars have observed that existing literature on self-management behaviors in metabolic bariatric surgery patients tends to either examine discrete domains of self-management in isolation or focus narrowly on the overarching construct of self-management, frequently relying on aggregate scores to assess behavioral proficiency (12, 17). Such variable-centered approaches overlook the inherent heterogeneity of these behaviors, thereby limiting insights into distinct behavioral patterns among patients after MBS. Single-domain investigations, in particular, fail to capture the complexity of multifaceted self-regulatory processes, further constraining our understanding of how different self-management components interact to influence surgical outcomes (18). A critical shortcoming of these approaches is their assumption of homogeneity within subgroups, which obscures qualitative differences between individuals. Conversely, Latent Profile Analysis (LPA)—a person-centered methodological framework—categorizes individuals by analyzing response patterns across multiple items, prioritizing maximal differentiation between latent classes while minimizing within-class variability (19). This approach further enables quantification of subgroup proportions within the broader population based on item-level responses, thereby revealing group disparities that remain undetected in variable-centered analyses (20). LPA has been widely adopted in psychological and social science research to identify distinct profiles of personal attributes. To date, however, there remains a paucity of updated research examining the heterogeneity of self-management behavior subgroups among patients after MBS.

In this study, we investigated the current status of self-management behaviors among post-metabolic bariatric surgery patients, analyzed subgroup differences and potential latent categories within the survey data, and examined factors influencing self-management outcomes. The aim of the study was to identify the profiles of patients after MBS in regards to varying levels of self-management behavior using LPA. By characterizing these profiles, this study seeks to provide empirical evidence and a theoretical basis for developing targeted intervention strategies to improve self-management among patients after MBS.

## Methods

### Participants

This cross-sectional study was conducted between July 2024 and October 2024 at obesity and metabolic surgery center, the Hospital. Participants were recruited via consecutive sampling from patients attending routine postoperative follow-up appointments. Eligibility criteria included:(1) Meeting the clinical indications for MBS, defined as a body mass index (BMI) ≥ 32.5 kg/m^2^, or a BMI ≥ 30 kg/m^2^ with obesity-related complications (e.g., metabolic syndrome, type 2 diabetes mellitus);(2) Aged ≥16 years;(3) At least 1 month post-MBS;(4) Ability to provide informed consent, with clear consciousness and no cognitive impairment. Exclusion criteria were: (1) Severe dysfunction of major organs (e.g., heart, liver, kidney);(2) Current pregnancy, lactation, or planned pregnancy within the next 6 months;(3) Severe gastrointestinal disorders;(4) Severe psychiatric disorders (including severe emotional or mental illness);(5) History of malignant neoplasms;(6) Use of weight loss medications or receipt of other weight-loss treatments within the past 3 months.

For latent profile analysis (LPA), prior research has demonstrated that the impact of sample size on statistical power is non-significant when other design parameters are controlled. Specifically, the sample-size adjusted Bayesian information criterion (SABIC) exhibits superior performance at sample sizes of *N* = 100 and *N* = 200 (21). Additionally, the Bayesian information criterion (BIC) performed exceptionally well even with small sample sizes. Notably, across all tested numbers of latent profiles, the average entropy was higher for *N* = 250 than for *N* = 1000; further, indices including the Lo-Mendell-Rubin likelihood ratio test (LMR), adjusted LMR, bootstrap likelihood ratio test (BLRT), BIC, and SABIC did not yield increased statistical power, particularly in larger sample sizes (22). In summary, given that sample size conditions exert only minimal effects on statistical power in LPA, a minimum sample size of 200 participants was chosen for the current study.

### Procedures

Before initiating formal data gathering, all researchers received standardized training to guarantee uniformity in carrying out the study procedures. Data were collected during participants’ 1-month postoperative outpatient follow-up visits. Prior to study enrollment, each participant was provided with a detailed explanation of the research objectives, methodologies, potential risks, and anticipated benefits. Written informed consent was obtained from all participants before they took part in the study. Participants completed a fully mandatory online questionnaire (i.e., no optional items included) distributed via the online platform “Questionnaire Star”. The entire process, encompassing the administration of the online survey, data storage, and data management, was executed with standardized protective measures that had received prior approval and documentation from the Ethics Research Committee of Zhongnan Hospital of Wuhan University (NO. 2023053K). Participants did not receive monetary compensation for their involvement, and all took part in the study entirely of their own free will.

### Measures

A total of 4 scales were used in this study, which were socio-demographic questionnaire, Bariatric Surgery Self-management Questionnaire, General Self-efficacy Scale and International Physical Activity Questionnaire. The socio-demographic data was collected by the research team through literature review, and the other 3 scales were based on previous studies. The reliability of the scales about the current study is stated in an explanation of each scale.

### Socio-demographic questionnaire

The self-designed questionnaire was used to obtain socio-demographic variables including age, gender, educational level, marital status, weight, height, employment condition, monthly income, and mode of operation. The questionnaire was filled out by patients independently and collected through the questionnaire website (Supplementary Material A1).

### Bariatric surgery self-management questionnaire (BSSQ)

The Chinese version of the BSSQ was employed to assess patients’ self-management behavior characteristics (23). It is a 33-item self-report scale (eg., “It took about 20–30 minutes for me to eat my meals”), including nine dimensions: eating behavior, choice of table ware, dietary water precautions, fluid intake, physical activity, dumping syndrome management, supplement intake, fruits, vegetables, whole grains intake, and protein intake. Each item is rated on a 4-point Likert scale (from 0 to 3). The overall scores ranges from 0 to 99, with higher scores suggesting the stronger self-management ability. In this study, the Cronbach's alpha was 0.867 (Supplementary Material A2).

### General self-efficacy scale (gses)

The Chinese version of the GSES was used to assess the level of self-efficacy (24). It is a single-dimension self-report measure with 10 items (e.g., “If you try hard, you can always accomplish a task efficiently”). Each item is rated on a 4-point scale (from 1 = totally wrong to 4 = absolutely wrong). The overall scores ranges from 10 to 40, with higher scores suggesting the higher level of self-efficacy. In this study, the Cronbach's alpha was 0.87 (Supplementary Material A3).

### International physical activity questionnaire (IPAQ)

The Chinese version of the IPAQ used to measure the level of physical activity in a population and included 27 items (eg., “Do you currently have a job or do any unpaid work outside your home” (25). It is a scale with five dimensions: career, traffic, household duties, leisure entertainment, and sit quietly. The exercise intensity of different activities was calculated in the form of a questionnaire and converted into metabolic equivalent (MET). Finally, the amount of activity was classified as “low-medium-high” by comparing the total metabolic equivalent value. In the study, the correlation coefficient of each group was above 0.7 (Supplementary Material A4).

### Data analysis

Data analyses were conducted with Mplus version 8.3 and IBM SPSS Statistics version 26.0. Since the data were collected through self-reported measures from a single source, we evaluated potential common method bias before conducting formal data analyses. For this purpose, the Harman's single-factor test was utilized via exploratory factor analysis (EFA), a well-established method for detecting common method bias in social science research. The EFA results showed that 25 factors with eigenvalues greater than 1 were extracted, together explaining 67.6% of the total variance. Importantly, the first factor individually explained 14.3% of the variance, which was markedly below the generally recognized critical cutoff of 40%. These results indicated that common method bias did not pose a significant problem in the current study.

Descriptive statistical analyses were conducted for all variables to describe the initial sociodemographic and clinical features of participants. Continuous variables were reported as mean ± standard deviation (SD), while categorical variables were expressed as frequencies (*n*) and percentages (%). LPA was utilized to identify distinct self-management behavior profiles using nine continuous variables. In order to ensure comparability across items, the total score of each scale was calculated as the average (i.e., determined by the mean of item scores within the scale). The optimal number of latent profiles was ascertained via multiple fit indices: Akaike information criterion (AIC), Bayesian information criterion (BIC), and adjusted Bayesian information criterion (aBIC) were employed to assess model fit, where lower values indicate better fit. The Lo-Mendell-Rubin (LMR) test and bootstrap likelihood ratio test (BLRT) were performed to examine whether a k-class model fitted significantly better than a (k-1)-class model; a significant p-value (*p* < 0.05) suggested that the k-class model was favored over the (k-1)-class model. Entropy was employed to evaluate classification accuracy, with higher values indicating greater precision (optimal threshold: >0.80). Differences in sociodemographic characteristics among latent profiles were analyzed using one-way analysis of variance (ANOVA) for continuous variables and the chi-squared (*x*^2^) test for categorical variables. Multinomial logistic regression was then conducted to explore the relationships between sociodemographic factors and latent profile membership.

## Results

### Descriptive and socio-demographic statistics

A total of 251 patients who met the inclusion criteria were initially recruited in this study, of which 9 were excluded for the following reasons: 3 had exacerbated obesity-related complications, 2 had weight regain exceeding 20% of their preoperative weight requiring reoperation, and 4 withdrew from the study due to personal reasons. A total of 242 valid samples were finally included, with an effective response rate of 96.41%, comprising 61 males (25.2%) and 181 females (74.8%). The mean age of the sample was 34.98 ± 9.43 years, with a range of 18–60 years. Preoperative median height and weight were 1.65 m and 105 kg, respectively. Most participants underwent sleeve gastrectomy as the surgical approach (*n* = 205, 84.71%). In terms of educational attainment, 232 participants (95.87%) had completed high school or higher education, 203 (83.88%) were in employment, and 142 (58.68%) were married. Regarding monthly income, 189 participants (78.1%) reported an income exceeding 3000 Chinese yuan (RMB). A total of 46 participants (19.01%) reported a smoking history, and 108 (44.63%) had a history of alcohol consumption. Additionally, most participants had a preoperative diagnosis of metabolic disease (91.3%, *n* = 221).

### Latent profile analysis

In this study, we used the 9 dimensions of the BSSQ as manifest variables to conduct LPA of self-management behaviors among patients after MBS. We tested potential profile models with 1–4 classes, as detailed in Table 1. The AIC, BIC, and aBIC values tended to decrease with an increasing number of profiles, while the entropy remained consistently above 0.8. According to the LMR test, the four-class model did not significantly improve model fit relative to the three-class model (*p* = 0.01). Considering both model fit metrics and the principle of parsimony, the three-class model was determined to optimally characterize the latent self-management behavior profiles. This model shows how patients scored on different parts of self-management, which can help create better support strategies for them.

**Table 1:** Fit statistics for the latent profile analysis.

Model	AIC	BIC	aBIC	Entropy	LMRT *p*-value	BLRT *p*-value
One-class	−1145.65	−1082.85	−1139.90	1		
Two-class	−2005.95	−1908.26	−1997.02	1	0.01*	0.01*
Three-class	−2122.42	−1989.84	−2110.29	1	0.01*	0.01*
Four-class	−2226.36	−2058.89	−2211.04	1	0.01*	0.01*

AIC, akaike information criterion; BIC, Bayesian information criterion; aBIC, adjusted Bayesian information criterion; LMR, Lo-Mendell-Rubin test; BLRT, bootstrap likelihood ratio test.

* *p* < 0.05. Boldface indicates the selected model.

**Figure 1:**
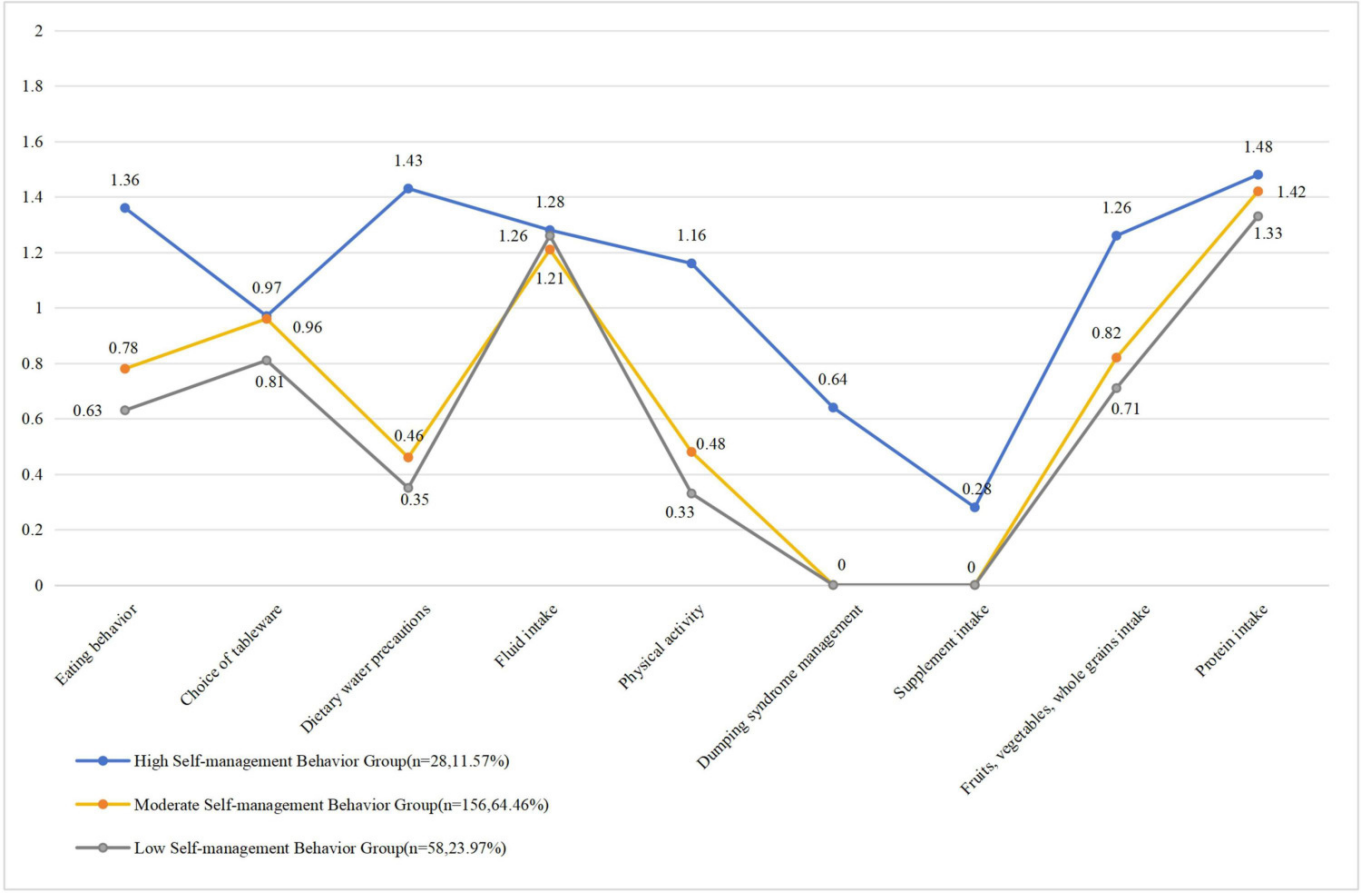
Latent profile for BSSQ mean values for the three-profile solution.

Statistical analyses revealed significant heterogeneity in self-management behaviors among patients after MBS, as evidenced by distinct mean score patterns across the three latent profiles (Table 2; Figure 1): Profile 1 showed higher aggregate scores across dimensions compared to Profiles 2 and 3; Profile 2 had slightly higher overall scores than Profile 3, except for scoring lower in the fluid intake dimension relative to Profile 3; and Profile 3 presented the lowest scores across all dimensions. Their key features are visualized in Figure 1. Profile 1 (*n* = 28, 11.57%), labeled the high self-management behavior group, was defined by the highest BSSQ scores and intensity of self-management behaviors. Profile 2 (*n* = 156, 64.46%), designated the moderate self-management behavior group, was characterized by a medium level of self-management practices. Profile 3 (*n* = 58, 23.97%), referred to as the low self-management behavior group, exhibited the lowest BSSQ scores and behavioral intensity. Among the three profiles, the high self-management group demonstrated higher scores in eating behavior, dietary water precautions, intake of fruits, vegetables, whole grains, and protein. The moderate and low self-management groups showed relatively higher scores in fluid intake and protein intake but lower scores in dietary water precautions. Notably, all three profiles shared lower scores in dumping syndrome management and supplement intake dimensions.

**Table 2:** Descriptive statistics for BSSQ variables that constituted the three profiles.

Variable	High self-management behavior group M(SD)	Moderate self-management behavior group M(SD)	Low self-management behavior group M(SD)	*F*
Eating behavior	1.19 (0.15)	0.81 (0.16)	0.82 (0.14)	79.312***
Choice of tableware	0.95 (0.39)	0.97 (0.12)	0.98 (0.09)	0.440
Dietary water precautions	1.41 (0.24)	1.01 (0.06)	0.47 (1.03)	805.099***
Fluid intake	1.27 (0.16)	1.41 (0.16)	1.33 (0.14)	14.322***
Physical activity	1.02 (0.35)	0.50 (0.18)	0.47 (0.18)	80.592***
Dumping syndrome management	0.62 (0.21)	0.01 (0.04)	0.01 (0.07)	608.374***
Supplement intake	0.13 (0.13)	0.00 (0.00)	0.00 (0.00)	121.931***
Fruits, vegetables, whole grains intake	1.17 (0.17)	0.91 (0.23)	0.86 (0.22)	20.520***
Protein intake	1.45 (0.26)	1.63 (0.17)	1.61(0.19)	11.774***

*** *p* < 0.001.

LPA was used to classify self-management behaviors among 242 patients who underwent metabolic bariatric surgery. Three latent profiles were identified: the high self-management behavior group (*n* = 28, 11.57%), the moderate self-management behavior group (*n* = 156, 64.46%), and the low self-management behavior group (*n* = 58, 23.97%).

### Predictor of latent profile membership

We carried out a one-way ANOVA and chi-squared test for three profiles of this study. The chi-squared test revealed significant differences between the three profiles regarding age (*F* = 15.93, *p* = 0.03), educational level(*x*^2^ = 7.83, *p* = 0.02; Table 3). Patients with high school or higher education were concentrated in the medium self-management behavior group (*n* = 152).

**Table 3:** Descriptive statistics in the full sample and each latent profile.

Variables	Total sample	High self-management behavior group	Moderate self-management behavior group	Low self-management behavior group	*F*/*x*^2^	*p*
Gender					2.39	0.30
Male	61 (25.21%)	7 (25%)	35 (22.44%)	19 (32.76%)		
Female	181 (74.79%)	21 (75%)	121 (77.56%)	39 (67.24%)		
Age M(SD)	34.98 (9.43)	30.57 (9.72)	35.72 (9.59)	35.1 (8.37)	15.93	0.03
Height, Median (Q1,Q3)	1.65 (1.6,1.7)	1.65 (1.62,1.73)	1.65 (1.6,1.7)	1.65 (1.6,1.7)	-	0.81
Weigh, Median (Q1,Q3)	105 (90.25, 130)	106.8 (96.82, 127.7)	104 (90, 129.1)	111.75 (93.95, 145)	-	0.45
Mode of operation					1.67	0.43
Sleeve gastrectomy	205 (84.71%)	26 (92.86%)	131 (83.97%)	48 (82.76%)		
Gastric bypass surgery	37 (15.29%)	2 (7.14%)	25 (16.03%)	10 (17.24%)		
Employment condition					3.66	0.16
Employed	203 (83.88%)	20 (71.43%)	133 (85.26%)	50 (86.21%)		
Not employed	39 (16.12%)	8 (28.57%)	23 (14.74%)	8 (13.79%)		
Educational level					7.83	0.02
Middle school/lower	10 (4.13%)	0 (0)	4 (2.56%)	6 (10.34%)		
High school/Higher	232 (95.87%)	28 (100%)	152 (97.44%)	52 (89.66%)		
Marriage status					5.13	0.08
Married	142 (58.68%)	11 (39.29%)	97 (62.18%)	34 (58.62%)		
Single/Divorced/Widowed	100 (41.32%)	17 (60.71%)	59 (37.82%)	24 (41.38%)		
Smoking history					3.56	0.17
Yes	46 (19.01%)	2 (7.14%)	30 (19.23%)	14 (24.14%)		
No	196 (80.99%)	26 (92.86%)	126 (80.77%)	44 (75.86%)		
Drinking history					5.12	0.08
Yes	108 (44.63%)	7 (25%)	75 (48.08%)	26 (44.83%)		
No	134 (55.37%)	21 (75%)	81 (51.92%)	32 (55.17%)		
Monthly income(RMB)					4.50	0.59
≤3000	53 (21.90%)	9 (32.14%)	32 (20.51%)	12 (20.69%)		
3001∼5000	73 (30.17%)	9 (32.14%)	45 (28.85%)	19 (32.76%)		
5001∼8000	82 (33.88%)	9 (32.14%)	55 (35.26%)	18 (32.14%)		
>8000	34 (14.05%)	1 (3.57%)	24 (15.38%)	9 (15.52%)		

We conducted multinomial logistic regression analyses to examine associations between patient characteristics (independent variables) and latent self-management profiles (dependent variables), with the moderate self-management group as the reference category (Table 4). The outcomes included total scores and dimension-specific scores on the BSSQ. Compared with unemployed patients, employed patients had a significantly lower likelihood of being classified in the high self-management group (OR: 0.07, 95% CI: 0.03–0.08) and a lower likelihood of being in the low self-management group (OR: 0.05, 95% CI: 0.02–0.07). Most employed patients (*n* = 133) were categorized in the moderate self-management group. Regarding smoking and alcohol consumption history: Patients with a smoking history were not significantly more likely to be in a specific self-management group (smoking history: OR = 0.75, 95% CI = 0.36–1.54). Patients with an alcohol consumption history were significantly less likely to be in the high self-management group compared to the moderate group (alcohol consumption history: OR = 0.36, 95% CI = 0.15–0.92).

**Table 4:** Multivariate multinomial logistic regression results predicting profile membership.

Variables	High self-management behavior group		Moderate self-management behavior group		Low self-management behavior group	
	OR(95%CI)	*p*	OR(95%CI)	*p*	OR(95%CI)	*p*
Gender						
Male	1.0 (0.30,3.35)	1	1.15 (0.45,2.93)	0.77	0.68 (0.25,1.89)	0.46
Female	Ref		Ref		Ref	
Age M(SD)	2.64 (1.03–6.81)	0.10	1.36 (0.58–3.21)	0.48	1.80 (0.71–4.57)	0.22
Height, Median (Q1,Q3)	1.32 (0.53–3.26)	0.66	0.97 (0.41–2.28)	0.94	0.97 (0.38–2.49)	0.95
Weigh, Median (Q1,Q3)	0.65 (0.26–1.62)	0.37	1.19 (0.15–2.80)	0.69	1.15 (0.45–2.95)	0.77
Mode of operation						
Sleeve gastrectomy	0.37 (0.08–1.81)	0.32	2.48 (0.55–11.12)	0.24	2.71 (0.55–13.30)	0.22
Gastric bypass surgery	Ref		Ref		Ref	
Employment condition						
Employed	18.21 (5.83–56.87)	<0.001	0.07 (0.03–0.18)	<0.001	0.05 (0.02–0.07)	<0.001
Not employed	Ref		Ref		Ref	
Educational level						
Middle school/lower	–	0.17	0.75 (0.31–1.81)	0.53	0.74 (0.28–1.96)	0.54
High school/Higher	Ref		Ref		Ref	
Marriage status						
Married	0.46 (0.18–1.15)	0.11	0.95 (0.25–3.62)	0.93	0.54 (0.13–2.18)	0.39
Single/Divorced/Widowed	Ref		Ref		Ref	
Smoking history						
Yes	0.32 (0.07–1.44)	0.18	0.75 (0.36–1.54)	0.05	0.24 (0.05–1.15)	0.08
No	Ref		Ref		Ref	
Drinking history						
Yes	2.44 (0.90–6.62)	0.10	0.36 (0.15–0.92)	0.02	0.41 (0.16–1.14)	0.10
No	Ref		Ref		Ref	
Monthly income(RMB)						
≤3000	0.61 (0.32–1.16)	0.13	0.78 (0.32–1.89)	0.59	0.48 (0.17–1.33)	0.16
3001∼5000	1.51 (0.81–2.82)	0.20	2.48 (0.85–7.20)	0.10	3.73 (1.21–11.45)	0.02
5001∼8000	1.89 (0.85–4.21	0.12	0.41 (0.18–0.94)	0.03	0.78 (0.39–1.55)	0.78
>8000	Ref		Ref		Ref	

OR, odds ratio, CI, confidence interval.

## Discussion

Using LPA, we stratified post-metabolic bariatric surgery (MBS) patients into three distinct subgroups based on self-management behavioral patterns: high, moderate, and low self-management profiles. The moderate self-management profile was the largest subgroup, comprising the majority of patients, which suggests that most post-MBS individuals exhibit intermediate self-management capacity. High self-management proficiency is critical for mitigating nutrient deficiencies, reducing complication risks, and preventing weight regain in post-MBS patients (26). Statistical analyses, including one-way ANOVA for continuous variables (age) and chi-square tests for categorical variables (educational attainment), revealed significant differences across the three subgroups. Specifically, elderly patients and those with lower educational backgrounds exhibited poorer postoperative behavioral adherence, consistent with prior observations (9, 24). Our findings are consistent with previous research showing that overall self-management adherence rates among patients after MBS typically range from 60% to 70%, indicating a moderate level of adherence. Common deficits include suboptimal dietary adherence (e.g., low protein intake, inadequate hydration) and poor physical activity adherence (14, 15). These domain-specific variations highlight the heterogeneity of self-management challenges. However, Wu et al. found that obesity patients after metabolic bariatric surgery exhibited high levels of self-management behavior, possibly due to the standardized multidisciplinary collaboration system established for post-MBS patients in recent years, which provides comprehensive and precise health management strategies to improve self-management behavior levels (27). Collectively, these results underscore the indispensable role of robust self-management capacity in sustaining long-term surgical outcomes (28, 29). Future investigations should prioritize exploring the underlying mechanisms driving interindividual differences in self-management behaviors and designing targeted intervention strategies tailored to subgroup-specific needs.

Contrary to previous studies demonstrating positive associations between self-management proficiency and weight-related anthropometric outcomes—whereby improved self-management behaviors correlate with greater reductions in body weight (11)—our study revealed no significant correlations between patient weight and the three self-management subgroups identified. This discrepancy may be attributed to two key factors: Firstly, the predominance of moderate self-management proficiency among the study cohort limits the variability necessary to detect meaningful associations. With most patients exhibiting intermediate self-regulatory abilities, the narrow range of behavioral performance may obscure potential relationships between self-management and weight outcomes. This is consistent with the findings of Bi et al. who found that when the distribution of self-management behaviors in the study population is too concentrated, the correlation between self-management and weight is significantly reduced (30). Secondly, persistent overweight status combined with suboptimal adherence to critical self-management domains—particularly dietary compliance and physical activity engagement—could mitigate the impact of self-management on weight regulation. In this study, even among the patients in the moderate self-management group, 38% failed to meet the postoperative dietary compliance standards, and 42% failed to meet the physical activity standards. This is similar to the findings of Sundgot-Borgen C et al., who discovered that even in patients with an average level of overall self-management behavior, the absence of these high levels of self-management behavior could hinder effective weight loss, thereby weakening the observable associations between different subgroup classifications and weight indicators (31, 32). Even among patients with moderate overall self-management, deficits in these high-priority behaviors may hinder meaningful weight reduction, thereby weakening observable correlations between subgroup classification and weight metrics.

Employment status has been identified as an independent prognostic factor for postoperative follow-up adherence among MBS patients, with employed individuals demonstrating higher follow-up adherence rates compared to unemployed or retired counterparts (12). Aligning with this, our findings revealed a higher proportion of gainfully employed patients within the high self-management cohort. This association may be attributed to two key advantages of employment: greater financial resources to support ongoing postoperative care and enhanced organizational skills to balance work responsibilities with follow-up appointments (33). Consistent with this economic dimension, the majority of patients in our high self-management group reported a monthly income exceeding 3,000 RMB, further supporting the link between socioeconomic stability and self-regulatory behavior. These results suggest that social factors may play a role in shaping self-management behavior after MBS, and future studies should explore the underlying mechanisms, such as the impact of social support networks or socioeconomic resources on adherence to post-MBS guidelines.

In addition to socioeconomic factors, self-efficacy—defined as a key psychological determinant of health behaviors—plays a pivotal role in shaping individual attitudes and actions. Notably, self-efficacy has been identified as a critical predictor of sustained post-surgical weight reduction following MBS (34). Patients with elevated self-efficacy exhibit stronger confidence in overcoming disease-related challenges, enabling them to adopt proactive health-seeking behaviors and maintain positive lifestyle modifications. This, in turn, contributes to improved long-term health outcomes and quality of life. Unlike the results of Wu et al.'s research on self-management behaviors, in this study, the employed population was more distributed in the moderate self-management behavior group. This might be related to the fact that the proportion of those engaged in high-intensity physical labor was relatively low in the sample of this study (27). Clinically, this study provides evidence for the self-management of post-MBS patients. Our study could help healthcare professionals analyze and identify subgroups of people at different levels by assessing self-management behavior in patients undergoing MBS. Health care providers should pay more attention to patients after MBS in moderate and low levels of self-management behavior. Personalized and precise management for different types of self-management behavior levels of post-MBS patients.

The present study has several limitations that warrant consideration. Firstly, the cross-sectional design limits our ability to establish causal relationships between self-management behaviors and weight outcomes. Longitudinal studies are needed to examine the dynamic changes in self-management behaviors and their impact on weight over time. Secondly, our study only included patients who were at least 1 month after MBS. The early postoperative period is critical for establishing self-management behaviors, and future studies should include patients at different time points post-surgery to examine the trajectory of self-management behaviors. Future studies should address these limitations to better understand the complex relationships between self-management behaviors and clinical outcomes after MBS. Thirdly, the study was conducted at a single medical center, which may limit the generalizability of our findings to other populations or settings. Future multi-center studies with more diverse samples are needed to confirm our results.

## Conclusions

To our knowledge, this study is to systematically characterize self-management behavior profiles among patients after MBS using latent profile analysis. We identified three distinct patterns of self-management behavior and found the moderate self-management group constituted the largest subgroup, exhibiting partial adherence to dietary guidelines and physical activity recommendations but lower engagement in follow-up care and supplement intake. Our findings provide a robust foundation for future longitudinal studies investigating the causal relationship between self-management behavior and long-term post-MBS outcomes, such as weight regain, metabolic control, and quality of life. Furthermore, our results emphasize the critical importance of conducting comprehensive pre-intervention assessments of self-management behavior to identify patients at risk of poor outcomes. Demographic variations in self-management profiles—including significant differences in age and educational level—highlight the need for targeted, culturally sensitive interventions. By tailoring interventions to specific patient subgroups, healthcare providers can optimize post-MBS outcomes and reduce disparities in care.

## Data Availability

The datasets presented in this study can be found in online repositories. The names of the repository/repositories and accession number(s) can be found in the article/Supplementary Material.
