# Supplementary material for: A latent profile analysis of self-management behavior among patients after metabolic bariatric surgery
Source: Front Health Serv. 2026 Mar 13;6:1774099. doi: 10.3389/frhs.2026.1774099 (PMC13036972; doi:10.3389/frhs.2026.1774099)
Supplement: Supplementary file 1 [file Datasheet1.pdf]

Appendix: Overview of the Questionnaires Used in the Study

Appendix 1. Social-Demographic Questionnaire

Note: This questionnaire was developed by the researchers to collect basic information about the participants.

Question	Item				
1. Gender	Male	Female			
2. Age	______ years				
3. Height	______ m				
4.Weight (1 week before surgery)	______ kg				
5. Mode of operation:	Sleeve gastrectomy	Gastric bypass	Other		
6. Employment condition	Freelance work	Administrative staff	Student	Worker	Courier retiree
7. Education level	Junior high school degree	High school degree	Junior college	Bachelor degree	Master's degree
8. Marital status	Married	Spinsterhood	Divorced		
9. Smoking history	Yes	No			

10. Drinking history	Yes	No		
11. Whether to quit smoking or drinking after surgery	Yes	No		
12. Preoperative disease(s) (e.g., hypertension, diabetes)	Yes	No		
13. Monthly income (RMB):	≤ 3,000	3,001 – 5,000	5,001 – 10,000	>10000
14. Payment method of hospitalization expenses:	Municipal medical insurance	Provincial medical insurance	Commercial insurance	Self-paying

Appendix 2. Chinese Version of the Bariatric Surgery Self-management Questionnaire (BSSQ)

Note: The BSSQ was used to assess self-management behaviors in the past week. The scale consists of 32 items across 9 dimensions, rated on a 4-point Likert scale from 0 (never) to 3 (always).

	Question	Item			
		Never	Seldom	Often	Always
Dietary Behaviors	1.I ate at least 5 meals/snacks during the day.				
	2.It took me about 20-30 minutes to finish a meal.				
	3.I chewed each mouthful of food into a puree/mush (more than 20 chews per mouthful), like baby food, before swallowing.				
	4.I checked for a feeling of fullness with every bite I took.				
	5.I ate slowly and put down my utensils or food between bites.				
	6.I took small sips of water or other drinks slowly and put them down between sips (approximately 2-3 ml per sip).				
Choice of Tableware	7.I used portion-control plates or measuring utensils instead of regular plates during meals.				
	8.I used baby-sized spoons and forks instead of regular-sized ones.				

Precautions for Eating and Drinking	9.I stopped eating immediately when I felt full or any discomfort.
	10.I avoided using a straw to drink.
Fluid Intake	11.I drank at least 2000 ml of fluid during the daytime.
	12.I only drank water, sugar-free beverages, skimmed milk, or 1% fat milk.
	13.I always carried water or a suitable beverage with me.
	14.I did not wait until I was thirsty to drink water.
	15.I checked my urine daily to ensure it was light yellow to clear, indicating good fluid intake.
	16.I avoided drinking liquids 30 minutes before and after meals to separate liquids from solid foods.
Physical Activity	17.In the past week, I exercised for at least 5 days, with activities lasting 30-60 minutes each day (e.g., walking, home equipment workout, gym session, video exercise class).
	18.I added some exercise to my daily activities, such as taking the stairs, or walking extra in the supermarket/mall before shopping.
	19.I included some weight-bearing exercises in my workout plan, such as lifting dumbbells, climbing stairs, or weightlifting.

Dumping Syndrome Management	20.I read nutrition labels to identify high-sugar foods.
	21.I avoided foods and beverages containing 15g (3 teaspoons) or more of sugar.
	22.I avoided foods where sugar (including glucose, maltose, dextrose, fructose, honey, molasses, corn syrup, brown sugar, sucrose, etc.) was listed as one of the first three ingredients on the label.
	23.I avoided foods with sugar alcohols (mannitol, sorbitol, xylitol, etc.) on the label, as these can cause stomach cramps and diarrhea.
Supplement Intake	24.I took a multivitamin with minerals tablet every day.
	25.I took Vitamin D along with 1000-1500 mg of calcium citrate or calcium carbonate daily.
	26.I took a B-complex vitamin supplement daily.
	27.I spaced my intake of vitamins and calcium pills by at least 4 hours to maximize absorption.
Fruits, Vegetables, and Whole Grains Intake	28.I ate at least 5 servings of fruits and vegetables daily.
	29.I mainly chose whole wheat bread, cereals, crackers, or coarse grains.
	30.I mainly chose brightly colored fruits and vegetables (yellow, green, red, orange, blue, purple).
Protein Intake	31.I supplemented with 60-80g of protein daily (protein powder, fish, eggs, chicken, beef, pork, etc.).

	32.I ate the protein on my plate first during meals and snacks.
	33.I read food labels and chose products with the highest protein and lowest sugar content.

Appendix 3. General Self-Efficacy Scale (GSES)

Note: Please indicate your agreement with the following statements regarding your general belief in your ability to handle various situations.(1 = Not at all true, 2 = Hardly true, 3 = Moderately true, 4 = Exactly true)

Question	Item			
	Not at all true	Hardly true	Moderately true	*Exactly true*
1. I can always manage to solve difficult problems if I try hard enough.				
2. If someone opposes me, I can find the means and ways to get what I want.				
3. It is easy for me to stick to my aims and accomplish my goals.				
4. I am confident that I could deal efficiently with unexpected events.				
5. Thanks to my resourcefulness, I know how to handle unforeseen situations.				
6. I can solve most problems if I invest the necessary effort.				
7. I can remain calm when facing difficulties because I can rely on my coping abilities.				
8. When I am confronted with a problem, I can usually find several solutions.				
9. If I am in trouble, I can usually think of a solution.				
10. I can usually handle whatever comes my way.				

Appendix 4. International Physical Activity Questionnaire – Long Form (IPAQ-SF), Last 7 Days

Note: This version assesses physical activity across multiple life domains. Heavy physical activity refers to the activity that requires you to spend a lot of effort to complete, and the breathing is significantly enhanced compared with the usual activity; Moderate-intensity physical

activity refers to the activity that requires you to spend moderate energy to complete and breathe slightly stronger than usual.

Section & Instructions	Question	Response Options
	1a.Are you currently engaged in paid work or unpaid work outside the home?	Yes
		No (Skip to PART 2)
	1b.During the last 7 days, on how many days did you do vigorous-intensity physical activities like heavy lifting, digging, or construction as part of your work? (Think about only those activities you did for at least 10 minutes at a time.)	______ days per week
		None (Skip to Question 1d)
	1c.How much time did you usually spend on one of those days doing vigorous-intensity physical activities at work?	___hours ___minutes per day
**PART 1: JOB-RELATED PHYSICAL ACTIVITY**<br>*Note:This section asks about your work. This includes paid work, farming, and other unpaid work you did outside your home. Do NOT include housework, which will be asked about in Part 3.*	1d.During the last 7 days, on how many days did you do moderate-intensity physical activities like carrying light loads as part of your work? (Do NOT include walking. Only activities for at least 10 minutes at a time.)	______ days per week
		None (Skip to Question 1f)
	1e.How much time did you usually spend on one of those days doing moderate-intensity physical activities at work?	____ hours ___ minutes per day

	1f.During the last 7 days, on how many days did you walk at work for at least 10 minutes at a time? (Do NOT include walking to/from work.)	______ days per week<br><br>None(Skip to PART 2)
	1g.How much time did you usually spend on one of those days walking at work?	______ hours ______ minutes per day
**PART 2: TRANSPORTATION PHYSICAL ACTIVITY**<br>*Note:This section asks about physical activity from transportation, such as commuting to work, shopping, etc.*	2a.During the last 7 days, on how many days did you travel by motor vehicle (e.g., train, bus, car, taxi)?	______ days per week<br><br>None (Skip to Question 2c)
	2b.How much time did you usually spend on one of those days traveling by motor vehicle?	______ hours ______ minutes per day
	2c.During the last 7 days, on how many days did you ride a bicycle for at least 10 minutes at a time?	______ days per week<br><br>None (Skip to Question 2e)
	2d.How much time did you usually spend on one of those days riding a bicycle?	_____hours ____minutes per day
	2e.During the last 7 days, on how many days did you walk for at least 10 minutes at a time? (Include walking for transportation.)	______ days per week<br><br>None (Skip to PART 3)

	2f.How much time did you usually spend on one of those days walking?	____hours ____minutes per day
	3a.During the last 7 days, on how many days did you do vigorous-intensity activities like heavy lifting or chopping wood in the yard or garden?	______ days per week<br><br>None (Skip to Question 3c)
	3b.How much time did you usually spend on one of those days doing vigorous-intensity activities in the yard or garden?	____ hours ____ minutes per day
**PART 3: HOUSEWORK, HOUSEHOLD, AND YARD MAINTENANCE ACTIVITY**<br>*Note:This section asks about household and yard work. Consider only activities done for at least 10 minutes at a time.*	3c.During the last 7 days, on how many days did you do moderate-intensity activities like carrying light loads or sweeping in the yard or garden?	______ days per week<br><br>None (Skip to Question 3e)
	3d.How much time did you usually spend on one of those days doing moderate-intensity activities in the yard or garden?	_____hours ____minutes per day
	3e.During the last 7 days, on how many days did you do moderate-intensity activities inside your home, such as washing windows, mopping floors, or vacuuming?	______ days per week<br><br>None (Skip to PART 4)
	3f.How much time did you usually spend on one of those days doing moderate-intensity activities inside your home?	____hours ____ minutes per day
**PART 4: RECREATION, SPORT, AND LEISURE-TIME PHYSICAL ACTIVITY**	4a.During the last 7 days, on how many days did you walk in your leisure time for at least 10 minutes at a time? (Do NOT	______ days per week

Note:This section asks about sports, exercise, and leisure activities done for at least 10 minutes at a time in your leisure time during the last 7 days. Do NOT include activities already reported above.	include walking already reported.)	
		None (Skip to Question 4c)
	4b.How much time did you usually spend on one of those days walking in your leisure time?	____ hours ____ minutes per day
	4c.During the last 7 days, on how many days did you do vigorous-intensity activities like running, swimming, or playing soccer in your leisure time?	______ days per week
		None (Skip to Question 4e)
	4d.How much time did you usually spend on one of those days doing vigorous-intensity activities in your leisure time?	____ hours ____ minutes per day
	4e.During the last 7 days, on how many days did you do moderate-intensity activities like playing table tennis or badminton in your leisure time?	______ days per week
		None(Skip to PART 5)
	4f.How much time did you usually spend on one of those days doing moderate-intensity activities in your leisure time?	______ hours ______ minutes per day
**PART 5: TIME SPENT SITTING**<br>*Note:This section asks about time spent sitting. Include time at work, at home, while studying, visiting friends, reading, or watching television. Do NOT include time spent sitting*	5a.During the last 7 days, how many were weekdays and how many were weekend days?	______ weekdays, ______ weekend days

in a vehicle.		
	5b.During the last 7 days, how much time did you usually spend sitting on a weekday?	______hours _____ minutes per weekday
	5c.During the last 7 days, how much time did you usually spend sitting on a weekend day?	______ hours ______ minutes per weekend day
